# Prevalence of helminth and coccidian parasites in Swedish outdoor cats and the first report of *Aelurostrongylus abstrusus* in Sweden: a coprological investigation

**DOI:** 10.1186/s13028-017-0287-y

**Published:** 2017-03-23

**Authors:** Giulio Grandi, Arianna Comin, Osama Ibrahim, Roland Schaper, Ulrika Forshell, Eva Osterman Lind

**Affiliations:** 1 Department of Microbiology, National Veterinary Institute (SVA), Ulls väg 2B, 75189 Uppsala, Sweden; 2 Department of Disease Control and Epidemiology, National Veterinary Institute (SVA), Ulls väg 2B, 75651 Uppsala, Sweden; 3 Bayer Animal Health GmbH, Monheim, Germany; 4 Bayer Animal Health AB, Solna, Sweden

**Keywords:** Feline parasites, *Toxocara cati*, *Aelurostrongylus abstrusus*

## Abstract

**Background:**

This study was performed in order to gather recent epidemiological data on feline endoparasites in Swedish cats. Faecal samples from 205 outdoor cats were collected by their owners and submitted to the National Veterinary Institute for analysis. The study population was comprised of cats with access to an outdoor environment and with no history of anthelmintic treatment within the last 3 months. Intestinal parasites were detected with a centrifugal flotation technique and Baermann larval sedimentation was performed to detect metastrongylid lungworms. Eggs, larvae and oocysts were identified morphologically by microscopic examination. The following information was collected from cat owners: breed, sex, age, anthelminthic medication last used, observation of cestode proglottids and residential address.

**Results:**

Endoparasites were detected in 25% of samples. Eggs of *Toxocara cati* were found in 21% of samples, followed by taeniid eggs (4%), oocysts of *Cystoisospora felis*/*C. rivolta* and capillarid eggs (both 1%). One cat tested positive for *Toxoplasma gondii*-like oocysts. Larvae of *Aelurostrongylus abstrusus* were detected in one cat, which is the first published observation of this parasite in Sweden.

**Conclusions:**

The occurrence of intestinal parasites is rather high in outdoor cats in Sweden, which could indicate the need for more intensive deworming routines in the population. Clinical practitioners should be aware of the possible occurence of *A. abstrusus* in Swedish cats when considering potential causes of respiratory problems in cats in the future.

## Background

A survey carried out in 2012 on the feline population in Sweden reported 1,159,000 cats, which is a slight decrease compared to a previous study performed in 2006 [1]. About 20% of them lived in households with children, that together with other categories, i.e. pregnant women and immunocompromised individuals are at high risk of being infected and develop diseases related to feline parasites [1]. Apart from the anecdotal cosmopolitan distribution of the most common feline endoparasitic helminths, like *Toxocara cati*, *Toxascaris leonina*, *Taenia (Hydatygera) taeniaeformis* and *Aelurostrongylus abstrusus* [2–4], little is known about the status of infection in outdoor cats in Sweden, since they are not routinely tested for parasitic infections. The majority of faecal samples submitted for parasitological examination comes from cats showing clinical signs such as expulsion of helminths and diarrhoea. Moreover, little is known about the deworming frequency, partly because most anthelmintics for cats are purchased without the involvement of a veterinarian, making it difficult to collect figures about common deworming practices. Moreover, it is difficult to quantify the use of fenbendazole for deworming since this drug is also frequently used in cats for (off-label) treatment/control of *Giardia* spp. infection.

Regarding ascarids, the Swedish Medical Products Agency recommends the first treatment to be carried out between the fourth and the sixth week of life and repeated every 2–4 weeks, depending on the substance, until the kittens are delivered to their new owners. The queen should be dewormed at the same time. Further treatments of adult cats against ascarids (and cestodes) are recommended when there is evidence of infection [5]. Since indoor cats are generally less exposed to the risk of helminth infections from the environment [6], the main goal of the present study was to investigate the prevalence of endoparasitic infections in outdoor cats. The other two aims were to carry out for the first time a large scale parasitological survey of metastrongylid lungworms in Sweden and to investigate potential risk factors related to animal management for the presence of parasites. Beyond the lack of any previous information, a deeper epidemiological study regarding *A. abstrusus* was needed due both to the reported presence of this parasite in bordering countries (Denmark and Norway, see [7, 8]) and to the relatively high frequency of findings of *Crenosoma vulpis* in Swedish dogs during the last years. In fact, between 9.4 and 9.7% of canine faecal samples analysed with the Baermann test (more than 500 samples have been tested yearly at the National Veterinary Institute (SVA) during the last 3 years) showed the presence of *C. vulpis* (SVA, unpublished data). This phenomenon addresses at least some geographical areas of Sweden as a favorable environment for the development of carnivore metastrongylid lungworm infection. The collection of information about antiparasitic treatments coupled to the sample collection represented also a chance to gather some information about deworming routines, e.g. used compounds and administration frequency.

## Methods

### Study population

A total of 205 cats coming from all regions of Sweden were included in the study. Information regarding the study was spread via social media (Facebook) and local Veterinary Clinics across Sweden. Households with one to three cats could participate in the study and faecal samples from a maximum of two individuals per household were submitted for analysis. Further inclusion criteria were that cats should have access to outdoor environment and no anthelmintic treatment should have been undertaken within 3 months prior to sampling. If these criteria were fulfilled, the cat owner received a submission kit with instructions and a referral form including relevant information on cats (i.e. breed, age, gender, neutering). A questionnaire was added to the referral form to get information on deworming practices. The owners had to reply to three questions: (a) “When was the last time you dewormed your cat and with which drug?” (b) “How often do you deworm your cat?” and (c) “Have you observed white parasitic fragments (i.e. cestode proglottids) around the anus or on the fur of your cat?”.

### Sample collection and parasitological examination

From each cat three samples from three different occasions within 1 week were collected directly from the ground or from the litter box and placed in individual plastic bags. The total sample volume should be at least 50 ml. Multiple samples were requested in order to increase the probability of finding parasites. Samples were stored at 4–6 °C until shipment to SVA, where they were analysed on the day of arrival. Gastrointestinal parasites were detected with a centrifugal flotation technique employing a flotation solution of saturated salt and 500 g/l sugar (specific gravity 1.28). Briefly, faeces from each of the three samples, totally between 3 and 5 g, were dissolved in the flotation solution, passed through a sieve (150 µm aperture), transferred to a Clayton-Lane centrifuge 15 ml tube. A glass coverslip 18 × 18 mm was placed on the tube that was then centrifuged at 214×*g* for 5 min in a Thermo Fisher Scientific—Sorvall ST40 centrifuge (Life Technologies Europe BV, Stockholm, Sweden) equipped with a swing out rotor [15, 16]. After centrifugation, the coverslip was transferred to a microscope slide and examined for parasites at a magnification of 100–400× by one of two experienced biomedical scientists. A minimum of 10 fields were carefully examined at 400×. The reading was done in a blinded fashion, i.e. without any information about the cats. Results were recorded semi-quantitatively as follows: no, few, low number, moderate number, high number and very high number of eggs/oocysts. This technique does not allow the detection of protozoan parasites like *Giardia* and *Cryptosporidium*. Since taeniid cestode eggs are not shed in the intestine but originate from disintegrating proglottids, flotation is not considered the best method for cestode diagnosis; for this reason, the observation of cestode proglottids by the owner was recorded as information to be used in the risk factor analysis.

Presence of metastrongylid lungworms was assessed with a modified Baermann larval sedimentation. Approximately 10 g of faeces were enclosed in cotton-gauze, placed in a glass funnel closed at the bottom extremity with metal clumps and incubated at least 24 h at 20 °C. The first 15 ml were collected then from the bottom of the Baermann apparatus and centrifuged at 214×*g* for 5 min. The sediment in the test tube was transferred onto microscope slides, mixed with a drop of Lugol’s iodine stain and examined at the magnification of 100×.

Parasitic elements (PE: eggs, oocysts, larvae) were identified morphologically according to [16, 17]. Eggs of *T. cati*, oocysts of *C. felis*/*C.rivolta* and larvae of *A. abstrusus* could be identified to species level based on their morphology and or size, while capillarid eggs were not identified to species level. Since no molecular analyses were employed, “small” coccidia were defined as *Toxoplasma*-like oocysts, even if this definition can include other parasites as *Hammondia* sp. Molecular identification performed in other cases of Swedish cats have shown that these oocysts belong to *Toxoplasma gondii* (SVA, unpublished results). Regarding taeniid eggs, different species are morphologically indistinguishable and since no polymerase chain reaction (PCR) was performed, species could not be determined and it cannot be excluded that some of them were eggs of *Echinococcus multilocularis*, even if it has been shown that are only rarely infected by this parasite and moreover in Sweden *E. multilocularis* eggs or DNA have only been detected in faeces from foxes [18, 19]. Based on previous data from necropsy studies performed on Swedish cats the likelihood that these eggs belong to *T. taeniaeformis* is very high.

### Statistical analysis

The study population was characterized according to (i) animal-level variables: gender (female vs. male), neutering (no vs. yes), age (≤1 year vs. >1 year old), breed (purebred vs. crossbred); (ii) temporal variables: season of testing (first, second, third of fourth quarter of the year); (iii) managerial variables: occurrence of last deworming treatment (never, <1 year before, ≥1 year before), deworming frequency (never, once/year, twice/year or more), and observation of cestode proglottids (no vs. yes).

To investigate the role of the above-mentioned variables as potential risk factors for the presence of feline endoparasites, a multivariate logistic regression analysis was performed. The selection of variables to include in the final model followed the approach proposed by Bursac et al. [20]. Briefly, the selection process began with a univariate analysis of each variable. Any variable having a *p* value for the Wald test exceeding 0.25 was selected as a candidate for the multivariate analysis. Subsequently, variables were checked for possible confounding and or effect modification, by looking at crude and adjusted odds ratios. At the end of this iterative process of deleting, refitting, and verifying, the model contained significant covariates, interactions and confounders. At this point any variable not selected for the original multivariate model was added back one at a time, to identify those which were not significantly related to the outcome but made an important contribution in the presence of other variables. The final multivariate model included the variables breed, observation of cestodes, deworming frequency and interactions between breed and observation of cestodes and between deworming frequency and observation of cestodes.

## Results

The majority (80%; n = 137) of cat owners in the present study (n = 171) sent faecal samples from one cat, while only 20% (n = 34) sampled two cats from the same household. Totally, 25% of the cats tested positive for parasitic faecal stages (Table 1). The most frequent parasites were ascarids, with eggs of *T. cati* being diagnosed in 20% of the samples, followed by 4% of samples positive for taeniid eggs and only 1% other endoparasites, oocysts of coccidia (*C. felis*/*C. rivolta*) and capillarid eggs. One cat tested positive for *T. gondii*-like oocysts. *A. abstrusus* larvae were observed in one cat from the county of Scania; the same cat shed capillarid eggs. This is the first published observation of *A. abstrusus* in Sweden.

**Table 1:** Results of the parasitological examination

Parasite	Samples (n)	% of positive samples	95% exact binomial confidence interval
*Toxocara cati*	43	20.98	[15.62–27.20]
Taeniid eggs	8	3.90	[1.70–7.54]
Capillarid eggs	3	1.46	[0.30–4.22]
*Cystoisospora felis*	1	0.49	[0.00–2.69]
*Cystoisospora rivolta*	1	0.49	[0.00–2.69]
*Toxoplasma gondii*-like	1	0.49	[0.00–2.69]
*Aelurostrongylus abstrusus*	1	0.49	[0.00–2.69]
Total positive samples	52	25.36	[19.56–31.90]
Total amount of collected samples	205	–	

Regarding intensity of infection, a variability was observed only for *T. cati*, those eggs were detected between few number and very high number, in most cases between moderate and high number (data not shown).

Regarding the drugs used to deworm cats (Table 2), 44% of the owners did not provide information regarding substances used to deworm the cats. As expected the two most commonly used drugs were the prescription-free ones (milbemycin + praziquantel and fenbendazole), used by 62% of the owners that provided this information. The third most commonly used product (used by 16% of the owners that provided information on deworming products) was a combination of emodepside + praziquantel, that is sold on prescription.

**Table 2:** Drugs used by cat owners included in the study at the latest deworming occasion

Drug	% of cats (n = 205)	% of households (n = 171)
Milbemycin + praziquantel	22.4 (n = 46)	20.5 (n = 35)
Fenbendazole	13.7 (n = 28)	14 (n = 24)
Pyrantel embonate + praziquantel	2.9 (n = 6)	2.9 (n = 5)
Praziquantel	3.4 (n = 7)	3.5 (n = 6)
Emodepside + praziquantel	8.3 (n = 17)	8.8 (n = 15)
Selamectin	1 (n = 2)	0.6 (n = 1)
Pyrantel embonate	5.4 (n = 11)	5.3 (n = 9)
Not specified/applicable	42.9 (n = 88)	44.4 (n = 76)

According to the univariate logistic analysis, cross-breed (vs. purebred) and observation of cestodes seemed potential risk factors for having endoparasites (Table 3). However, results of the multivariate logistic analysis—which is more comprehensive since it allows to consider several variables at the same time and to correct for possible confounding and interactions—showed that none of the investigated variables were significant risk factors for the study population.

**Table 3:** Characterization of the study population: number, proportion, 95% exact binomial confidence intervals (CI) and results of univariate logistic analyses of the parasite-positive cats by the main variables (outcome variable = presence of parasites yes/no)

Variable	Trait	Number of positive samples	Number of samples	Proportion of positive samples	Exact binomial 95% CI	Wald test	p value
Gender	Female	21	96	0.22	[0.14–0.31]	1.20	0.28
	Male	31	109	0.28	[0.20–0.38]		
Neutering	No	31	127	0.24	[0.17–0.33]	0.16	0.69
	Yes	21	78	0.27	[0.18–0.38]		
Age	<1 year	7	22	0.32	[0.14–0.55]	0.12	0.73
	≥1 year	45	180	0.25	[0.19–0.32]		
Breed	Purebred	7	50	0.14	[0.06–0.27]	4.60	*0.032*
	Crossbred	44	148	0.30	[0.23–0.38]		
Testing period	Jan–Mar	12	49	0.24	[0.13–0.39]	1.30	0.73
	Apr–Jun	6	20	0.30	[0.12–0.54]		
	Jul–Sep	6	33	0.18	[0.07–0.35]		
	Oct–Dec	28	103	0.27	[0.19–0.37]		
Observation of proglottids on fur	No	21	122	0.17	[0.11–0.25]	11.2	*0.0008*
	Yes	30	77	0.39	[0.28–0.51]		
Last deworming treatment	Never	2	13	0.15	[0.02–0.45]	1.60	0.44
	<1 year before	29	95	0.31	[0.21–0.41]		
	≥1 year before	16	65	0.25	[0.15–0.37]		
Deworming frequency	Never	11	53	0.21	[0.11–0.34]	2.50	0.25
	Once/year	21	83	0.25	[0.16–0.36]		
	Twice/year or more	19	56	0.34	[0.22–0.48]		

Italic values indicate p < 0.05

## Discussion

The scope of this study was to investigate the occurrence of parasites in privately owned outdoor cats by faecal analyses. The results confirm that *T. cati* and taeniid cestodes are the most common feline endoparasites in Sweden. The occurrence was higher than previously thought—higher than in samples analysed within the routine diagnostics, where 5% of the cats shed helminth eggs (SVA, unpublished data, 2015–2016). Furthermore, it was assessed that none of the tested animal- and managerial-level variables was a significant risk factor for the presence of such parasites.

Cats can acquire infection by *T. cati* by different routes, either by ingesting infective eggs from the environment or ingesting larvae contained in rodents and other paratenic hosts or by ingestion of larvae passed in the milk by the queen (transmammary infection). Even if it has not been assessed which one of these routes is the most frequently involved in the transmission of the parasite, based on previous studies [2] it seems that ingestion of larvae present in paratenic hosts linked to hunting behavior of cats is considerably significant.

Regarding the most common cestode parasite of cats (*T. taeniaeformis*), cats become infected exclusively by ingesting the metacestode stage (strobilocercus) that develops in several species of rodents and potentially even in lagomorphs that have acquired infection through the ingestion of taeniid eggs [2].

In the case of coccidia, cats can become infected both by direct ingestion of sporulated oocysts or by ingestion of tissues of paratenic hosts containing coccidian sporozoites and usually infection is more common in kittens [2]. Since the cat population considered in the present study is biased towards adult cats, it is not surprising that these parasites occurred only in few animals.

The only results available from coprological analyses in Swedish cats come from a published summary of the results from 1371 samples submitted to SVA between the years 1958 and 1970 [9]. In comparison to our study, a similar amount of eggs of *T. cati* (19.4%) was detected whereas a higher amount of taeniid eggs (9.1%) and oocysts of *Cystoisospora* spp. (4%) were recorded. Moreover, eggs of several other helminths that were not recorded in the present study (*Toxascaris leonina*, *Uncinaria stenocephala*, *Cryptocotyle linguae*, *Opistorchis felineus*, *Diphyllobotrium latum*, *Dipylidium caninum*) were found, indicating that new available anthelmintics as well as improved deworming routines may have reduced the spectrum of parasitic infections in cats during the last decades. However, data collected from diagnostic routine cannot be regarded as representative of a general cat population.

Few other data are available regarding endoparasitic infections in Swedish cats. Two necropsy studies have been previously carried out in Sweden on a random population of cats. In the first one—performed on 83 cats from the area of Stockholm, Sweden [10]—19.3% of the cats were infected by *T. cati* and 9.6% of the cats were infected by *T. taeniaeformis*. One of the cats was infected by *Mesocestoides lineatus*. In the second study—performed on 100 cats from the county of Halland, Sweden [11]—61 and 31% of outdoor cats (n = 70) were infected with *T. cati* and *T. taeniaeformis*, respectively. Moreover, *Ancylostoma tubaeforme* was found in one cat and *Eucoleus aerophilus* in another. Even though these results come from necropsy studies, where more data on the occurrence of immature stages, species identification and intensity of infections can be recorded, both studies are in agreement with present observations, i.e. that *T. cati* and taeniid cestodes are the most common feline parasites in Sweden.

Analysis by a concentration McMaster technique of faecal samples (n = 95) from a population of necropsied Danish cats showed a much higher prevalence of parasitic infection (77.9%) in comparison to the present study. Eggs of *T. cati* were the most common (69.5%), followed by capillarid type eggs (16.8%), taeniid eggs (9.5%), oocysts of *C. felis* (2.1%) and strongyle type eggs (1.1%) [12]. A possible explanation for such high prevalence values can be the characteristics of the sampled population (92 of 99 cats examined by necropsy were feral). Another survey performed by flotation of faecal samples of 719 Danish shelter cats showed prevalence figures similar to our study (35.6 and 3.5% of cats infected by *T cati* and *T. taeniaeformis*, respectively, followed by lower prevalence of coccidia and *E. aerophilus*), but a slightly higher variety of parasites (*T. leonina* and *U. stenocephala* were found) [13].

Another recent survey carried out in Finland on faecal samples from 411 cats (63.5% with access to outdoor environment), also showed that the most common parasites were *T. cati* and *T. taeniaeformis* (5.4 and 1.7% of cats were infected, respectively). *T. leonina* and C. *felis* were found in few animals; *Giardia* sp. was detected in 3.2% of the cats using an ELISA kit. Outdoor cats showed a higher prevalence of infection for *T. cati* (7.28%) than indoor cats (2.50%), an observation that supports the higher exposure to infection of this class of animals. Also, cats receiving homemade food in the diet, as well as cats from rural areas showed higher risk of being infected by *T. cati*. In agreement with the present study, also cats where the owners had seen proglottids and non-purebred cats had a higher chance of being infected by *T. cati* [14].

Based on the present results clinicians should be aware that cats with access to outdoor environment can harbour feline ascarids as well as taeniid cestodes, and therefore an implementation of deworming routines is needed. Our suggestion is that a regular, monthly deworming of outdoor cats with substances active against nematodes and cestodes should be performed during the “hunting season”, i.e. whenever they have access to prey.

Regarding feline metastrongylid lungworms, this is the first published observation of their occurrence in Sweden. The cat that was shedding *A. abstrusus* larvae in the present study was a five-and-a-half-year-old female that was born on a farm and had never travelled abroad. Moreover, she had been dewormed in May 2014 (6 months before the time of sampling) with a product containing milbemycin oxime and praziquantel. Cats can become infected by *A. abstrusus* by ingesting third larval stages of the parasite that develop into several molluscan intermediate hosts; several paratenic hosts have been identified (rodents, birds, reptiles). It is unclear how often infection is due to direct ingestion of intermediate hosts rather than ingestion of paratenic hosts. According to recent studies infective larvae can even be released into the environment by snails, thus increasing the chances of cats to become infected [3]. The present finding is not surprising since the presence of *A. abstrusus* in Norway was documented long ago [8] and since this parasite is relatively common in Denmark [12]. In a recent Danish study, the prevalence of *A. abstrurus* in 147 outdoor cats from Zealand, Møn and Falster regions ranged between 13.6 and 15.6% depending on the method employed; in the Southern regions prevalence peaked to 72.7% [12]. It is difficult to say if *A. abstrusus* has been recently introduced into Sweden but probably this parasite has been unnoticed until now because of its low prevalence in Swedish cats, its uneven geographical distribution and because of the fact that few cats are tested for metastrongylid lungworms and common flotation methods do not allow detection of this parasite.

Even if more data is needed to assess the importance of feline zoonotic endoparasites as a source of human infections it can be assumed that the presence of such parasites (*T. cati* and probably *T. gondii*) can represent a potential source of environmental contamination. This information should be taken in account by all healthcare professionals. Faecal analysis could be useful for diagnosing patent *T. cati* infections. Taeniid cestode eggs are more difficult to detect by flotation since they generally are not liberated from the proglottids.

It is desirable that in the near future figures from necropsies of outdoor feral cats, as well a more widespread testing of cats for metastrongylid lungworms will provide a more detailed picture of the parasitic populations of Swedish cats, both in terms of intensity and of variety.

## Conclusions

The results indicated that outdoor cats in Sweden are regularly exposed to endoparasitic infections and the predominant role of *T. cati* was confirmed, followed by taeniid parasites. Current Swedish deworming recommendations assume that the prevalence of feline parasites in adult cats in Sweden is quite low, a statement that according to the present study is not true in the case of cats with outdoor access. Such cats—including adult ones—should receive regular anthelmintic treatments whenever they access to prey or whenever parasites are observed. Demonstrated presence of feline *A. abstrusus* in Sweden should be also taken in account by practitioners as one of the causes of respiratory signs.
